# Diagnostic Delay of a Vaginal Foreign Body: A Case Report

**DOI:** 10.7759/cureus.71259

**Published:** 2024-10-11

**Authors:** Rajamaheswari N, Harini Sivamani, Ananthakrishnan Sivaraman, Manoj Tummala, Sivaraman PB

**Affiliations:** 1 Urogynecology, Advanced Centre for Urogynaecology, Chennai, IND; 2 Obstetrics and Gynecology, Advanced Centre for Urogynaecology, Chennai, IND; 3 Uro-oncology, Chennai Urology and Robotics Institute, Chennai, IND; 4 Urology, Chennai Urology and Robotics Institute, Chennai, IND

**Keywords:** female, foreign body, urinary infection in female children, urogenital symptoms, vagina, vaginal spotting, vulvovaginitis, young girl

## Abstract

This report presents the case of vaginal foreign body (FB) in a seven-year-old girl with recurrent genitourinary symptoms that went unrecognized for two years. Despite thorough evaluation including MRI, cystourethroscopy, and several consultations with various specialists, the cause could not be diagnosed. Recurrent symptoms forced her to approach our center. Repeat cystourethroscopy with vaginal examination under anesthesia (VEUA) enabled the detection of the FB in the vagina. Removal helped achieve permanent relief. VEUA is crucial and is a test not to be missed in the evaluation protocol of vaginal FB. This report establishes that even MRI and cystourethroscopy without VEUA can miss the diagnosis.

## Introduction

Vaginal foreign body (FB) in children is uncommon, but diagnostic delay is not that uncommon [1,2]. Often, FB in the vagina goes unrecognized despite recurrent episodes of vulvovaginitis with diverse genitourinary symptoms. Vaginal FB commonly presents with vaginal discharge, vaginal spotting, lower urinary tract symptoms, and pain in the lower abdomen [3,4]. Vaginal FB is mostly observed between the second and ninth years of life [5] and is frequently encountered in children with intellectual disability. The common object identified is a wad of toilet paper, which is found in up to 80% of cases [6]. Others reported objects are safety pins, pencils, hairpins, etc., inserted out of curiosity [7].

This report presents the case of vaginal FB with recurrent genitourinary symptoms which went unrecognized on several consultations by various specialists over a period of two years.

## Case presentation

A seven-year-old girl presented with a two-year history of recurrent foul-smelling vaginal discharge, vulvovaginal itching, burning micturition, dysuria, urgency, urge incontinence, and spotting at the end of micturition. These symptoms persisted despite intermittent antibiotic treatment as part of conservative management. She had constipation, and her urinary frequency was normal (five during the day and one at night). She was suspected of a delay in developmental milestones. The girl lived with her parents, and there was no history suggestive of sexual abuse. In the past two years, she resorted to multiple consultations with a gynecologist, urologist, and pediatric urologist. Prior to her visit to our center, she underwent a thorough evaluation by various specialists with ultrasonogram of the kidney, ureter, and bladder (USG KUB), pelvic X-ray scan, MRI, and cystourethroscopy. However, the cause could not be identified.

In view of persisting symptoms, parents continued to seek help and finally approached our center. Physical examination including the neurological system was normal. On local examination, redness of the vulva, minimal vulval excoriation, and vaginal discharge were noted. The provisional diagnosis of vaginitis due to estrogen deficiency, infective vaginitis, and FB vagina was considered. The fact that the child had persistent vulvovaginitis with genitourinary symptoms for two years in spite of evaluation and treatment carried out many times by various specialists tipped off the possibility of vaginal FB.

**Figure 1:**
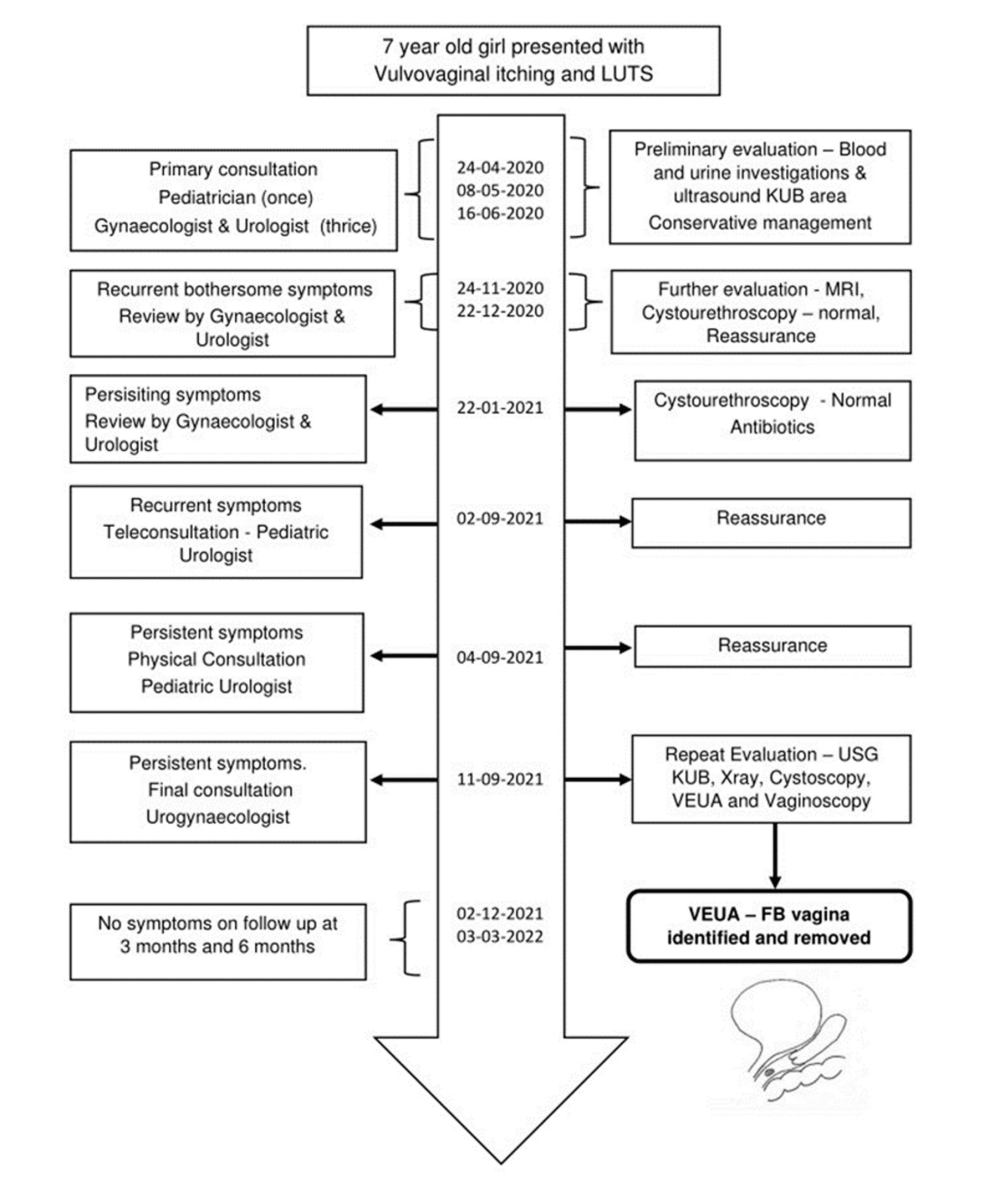
Timeline of management LUTS, lower urinary tract symptoms; KUB, kidney, ureter, and bladder; VEUA, vaginal examination under anesthesia

Further evaluation at our center revealed normal blood and urine parameters. USG KUB imaging and uroflowmetry were normal. She was subjected to repeat cystourethroscopy and vaginal examination under anesthesia (VEUA). Cystoscopy was normal, and VEUA revealed a brownish free-lying material (3 x 2 cm) in the lower vagina, which was removed (Figure 1).

**Figure 2:**
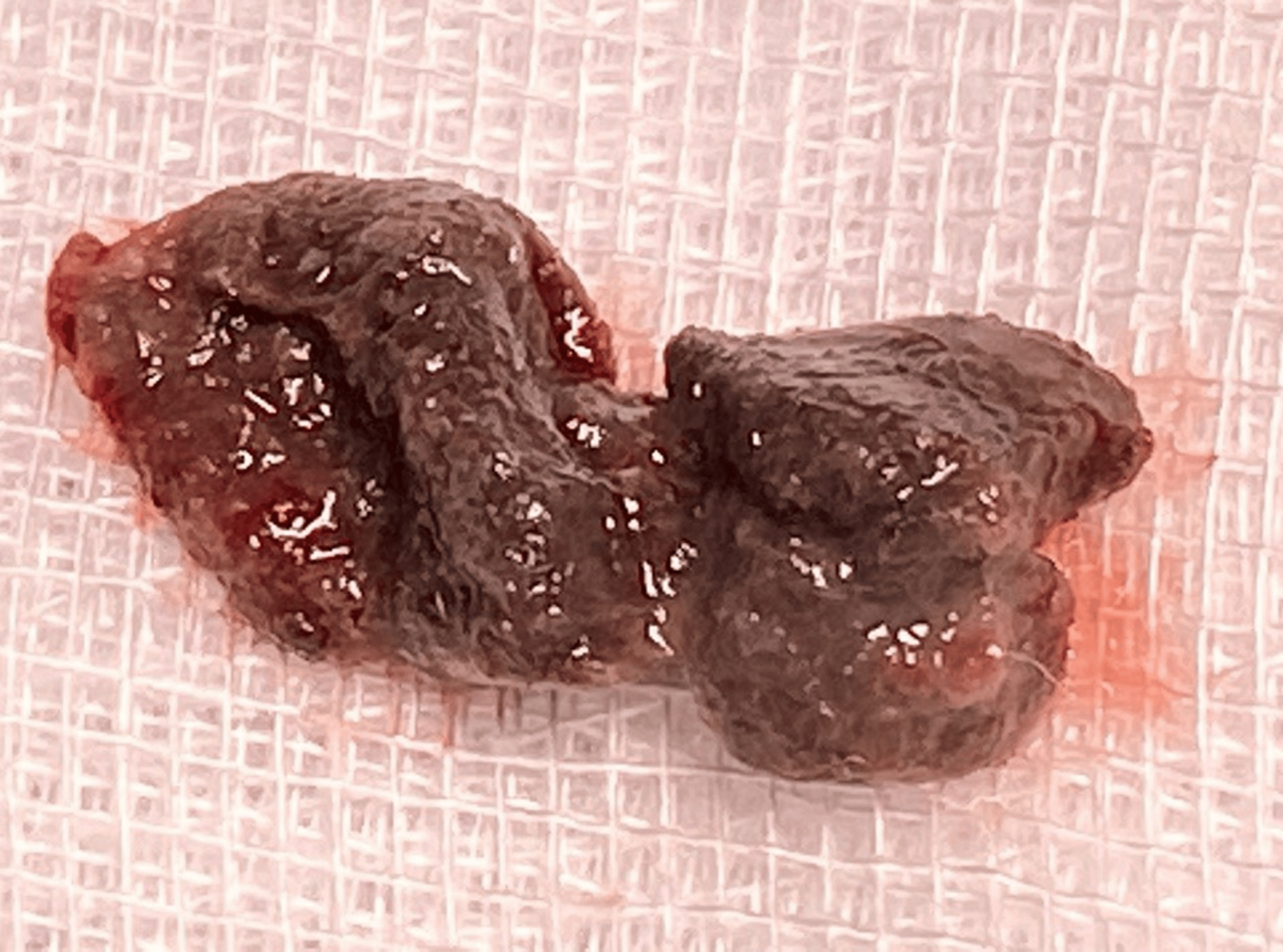
Vaginal foreign body

Vaginoscopy revealed a congested vagina without ulceration or growth. Histopathological examination of the “free lying tissue from the vagina” showed FB with granular necrotic debris, refractile structures, and bacterial colonization. The child was reviewed at three and six months after the removal of FB and was completely free of symptoms. Removal of FB provided definitive therapeutic relief (Figure 2).

## Discussion

Persistent vulvovaginal symptoms in children, as in this case, should raise the suspicion of vaginal FB. The possibility of sexual abuse should be considered if a child has persistent or recurrent vulvovaginal symptoms even in the absence of a history of sexual abuse. Leading questions to obtain details about sexual or physical abuse often fail as children intentionally or unintentionally fail to disclose FB insertion into the vagina as in this case.

Vaginal FB can lead to vaginitis, ulceration, and urinary or fecal fistulae. [8] Ascending infection can lead to uterine infection, salpingitis, peritonitis, and acute abdomen. Hence, female children with persistent or recurrent vulvovaginitis with or without urinary symptoms demand thorough evaluation and continuous surveillance. Female children with suspected vaginal FBs are generally evaluated with pelvic ultrasonography, plain X-ray of the pelvis, vaginography, and MRI to confirm the diagnosis. MRI is considered the best technique for recognizing intra-vaginal FB [9]. However, a VEUA identified the vaginal FB in this case, even though MRI has failed to detect it.

Removal of vaginal FB in children is simple and easily carried out without any advanced medical equipment, though the use of the illuminated endoscope has been described. Wang et al. used continuous flow vaginoscopy to detect and remove vaginal FB [10]. After the removal of the FB, the vaginal wall heals by itself, as witnessed in the current report. A multidisciplinary approach involving a pediatrician, gynecologist, urogynecologist, and pediatric psychologist is recommended for complete cure and follow-up. The provision of a child support team and psychosocial services can help identify and eliminate the root cause and ensure continuity of care [11].

This case emphasizes that persistent vulvovaginitis with genitourinary symptoms in a female child should never be ignored and that a high index of suspicion for a vaginal FB is necessary. As in this case, even if the patient’s all past evaluations (including MRI) fail to recognize the reason, the clinician should pursue the evaluation till the cause is unearthed and the problem is solved. This case establishes that all the other evaluation (including MRI) modalities except VEUA failed to detect the cause. It is imperative that the treating physician should include VEUA as part and parcel of the evaluation protocol of persistent or recurrent vulvovaginitis in children.

The persistence of genitourinary symptoms, though not life-endangering, motivated the parents to seek consultation with several specialists of different domains (pediatrician, gynecologist, urologist) in their native state. Failure to find a solution forced the parents to seek help in different centers across the country. Despite the perseverance of the parents, the diagnosis got delayed for two years due to the anxiety-triggered medical tourism indulged by the parents resulting in a lack of continuity in case follow-up, which delayed the treatment in general and by the primary specialist.

## Conclusions

Vaginal FB is likely to go unrecognized unless there is a high index of suspicion. A systematic and thorough evaluation is essential. Continuous surveillance with constant vigilance should be implemented. A clinician should pursue the search till the cause is disclosed. It is crucial to include VEUA in the evaluation protocol of female children presenting with persistent or recurrent vulvovaginitis and genitourinary symptoms.

## References

[1] Di Meglio G (1998). Genital foreign bodies. Pediatr Rev.

[2] Striegel AM, Myers JB, Sorensen MD, Furness PD, Koyle MA (2006). Vaginal discharge and bleeding in girls younger than 6 years. J Urol.

[3] Dahiya P, Sangwan K, Khosla A, Seth N (1999). Foreign body in vagina--an uncommon cause of vaginitis in children. Indian J Pediatr.

[4] Chinawa J, Obu H, Uwaezuoke S (2013). Foreign body in vagina: an uncommon cause of vaginitis in children. Ann Med Health Sci Res.

[5] Stricker T, Navratil F, Sennhauser FH (2004). Vaginal foreign bodies. J Paediatr Child Health.

[6] Closson FT, Lichenstein R (2013). Vaginal foreign bodies and child sexual abuse: an important consideration. West J Emerg Med.

[7] Padmavathy L, Ethirajan N, Rao LL (2004). Foreign body in the vagina of a 3(1/2)-year-old child: sexual abuse or a childish prank?. Indian J Dermatol Venereol Leprol.

[8] Biswas A, Das HS (2002). An unusual foreign body in the vagina producing vesicovaginal fistula. J Indian Med Assoc.

[9] Kihara M, Sato N, Kimura H, Kamiyama M, Sekiya S, Takano H (2001). Magnetic resonance imaging in the evaluation of vaginal foreign bodies in a young girl. Arch Gynecol Obstet.

[10] Wang CW, Lee CL, Soong YK (1996). Hysteroscopic extraction of a vaginal foreign body in a child. J Am Assoc Gynecol Laparosc.

[11] Herman-Giddens ME (1994). Vaginal foreign bodies and child sexual abuse. Arch Pediatr Adolesc Med.

